# Magnitude and relevance of change in health-related quality of life in patients with vascular malformations treated with sirolimus

**DOI:** 10.3389/fmed.2023.1155476

**Published:** 2023-04-20

**Authors:** Veroniek E. M. Harbers, Frédérique C. M. Bouwman, Ingrid M. P. van Rijnsoever, Bas H. Verhoeven, Carine J. M. van der Vleuten, Leo J. Schultze Kool, Peter C. J. de Laat, Chantal M. A. M. van der Horst, Wietske Kievit, D. Maroeska W. M. te Loo

**Affiliations:** ^1^Medical Imaging, Radboud University Medical Center, Nijmegen, Netherlands; ^2^Radboudumc Center of Expertise HECOVAN, Amalia Children's Hospital, Radboud University Medical Center, Nijmegen, Netherlands; ^3^Department of Surgery, Radboud University Medical Center, Nijmegen, Netherlands; ^4^Department of Pediatrics, Amalia Children's Hospital, Radboud University Medical Center, Nijmegen, Netherlands; ^5^Department of Dermatology, Radboud University Medical Center, Nijmegen, Netherlands; ^6^Members of the Vascular Anomalies Working Group (VASCA WG) of the European Reference Network for Rare Multisystemic Vascular Diseases (VASCERN), Paris, France; ^7^Department of Pediatric Oncology, WEVAR-Team, Rotterdam Erasmus MC-Sophia, Rotterdam, Netherlands; ^8^Department of Plastic Reconstructive and Hand Surgery, AVA-Team, Amsterdam University Medical Center, Amsterdam, Netherlands; ^9^Health Technology Assessment, Department for Health Evidence, Radboud University Medical Center, Nijmegen, Netherlands; ^10^Department of Pediatric Hematology, Amalia Children's Hospital, Radboud University Medical Center, Nijmegen, Netherlands

**Keywords:** vascular malformation, quality of life, sirolimus (rapamycin), SF-36: 36-item short form health survey, PedsQL: pediatric quality of life inventory

## Abstract

**Introduction:**

Vascular malformations are rare congenital anomalies of the vascular system, which can involve the capillaries, veins, arteries, lymphatics, or a combination of vessel types. Patients with vascular malformations experience an impaired health-related quality of life (HRQoL) because of their symptoms (e.g., pain, swelling, and bleeding) and psychosocial distress. Sirolimus is an effective drug used in the medical treatment of these patients; however, relatively little is known about the effect of sirolimus on specific changes in the HRQoL domains and its magnitude.

**Methods:**

The magnitude of change (effect size) following intervention is more informative to clinical practitioners than statistically significant but clinically unimportant changes; therefore, this study aimed to examine the magnitude and meaningfulness of change in the HRQoL of children and adults with vascular malformations following sirolimus treatment using low target levels.

**Results:**

In total, 50 patients with vascular malformations (19 children, 31 adults) were included in this study. These patients experienced a lower HRQoL than the general population, with the adults reporting a significantly lower score in almost all domains. A 6-month sirolimus treatment improved the HRQoL in 29 patients, including 77.8% of the children (Pediatric Quality of Life Inventory score [PedsQL]) and 57.7% of the adults (Short Form 36 [SF-36]). The effect sizes of sirolimus for each SF-36/PedsQL domain ranged from 0.19 to 1.02. The clinically relevant moderate magnitude of changes was seen in the domains of the children's reports: “Physical functioning” and “Social functioning” and in the domains of the parent reports: “Social functioning,” “School functioning,” and “Psychosocial.” A high-magnitude change was seen in the domains “Emotional functioning” and “Psychosocial” in the children's reports and “Physical functioning” in the parent reports. In addition, the moderate magnitude of changes was also seen in the adults SF-36: in all domains except for “Role limitations—physical problems,” “Role limitations—emotional problems,” and “General health perception.”

**Conclusion:**

We believe this is the first study showing the magnitude of change in HRQoL after sirolimus treatment in patients with vascular malformations. Before treatment, these patients experienced an impaired HRQoL compared with the general Dutch population. A 6-month sirolimus treatment with low target levels led to moderate-to-high clinically relevant changes in multiple domains, which significantly improved the HRQoL.

**Clinical trial registration:**

https://clinicaltrials.gov/ct2/show/NCT03987152?cond=Vascular+Malformations&cntry=NL&city=Nijmegen&draw=2&rank=1, identifier: NCT03987152.

## Introduction

Vascular malformations are rare congenital anomalies of the vascular system that grow proportionally with age. These malformations can involve the capillaries, veins, arteries, lymphatics, or any combination of these (1, 2). Of these, venous and lymphatic malformations are categorized as low-flow vascular malformations. Most of the frequently found somatic mutations in low-flow vascular malformations occur in genes involved in the mammalian target of rapamycin (mTOR) pathway; for example, PIK3CA and TEK/TIE-2 mutations lead to a gain of function and increased activity of mTOR (3–7). Patients experience symptoms of pain, swelling, bleeding, ulcerations, leakage, thrombotic complications, disfigurement, functional impairment, and psychosocial distress, which affects their health-related quality of life (HRQoL) (8–12).

The treatment options for low-flow vascular malformations are conservative, including compressive hosiery, analgesics, anti-inflammatory or anti-coagulation drugs, intralesional sclerotherapy or embolization, and surgery (13). Sclerotherapy alone or in combination with surgical resection is frequently applied in most vascular malformations (14, 15). Unfortunately, treatment is challenging and not always successful and can leave patients with a high clinical burden and subsequently a reduced HRQoL (16).

The treatment of pain and other symptoms in patients with vascular malformations can improve their HRQoL. Several studies have shown that sirolimus, an mTOR inhibitor, can reduce pain and, in some cases, may reduce the size of the vascular malformation (17–23). The reduction in symptoms due to sirolimus may improve the HRQoL. To measure HRQoL in patients with vascular malformations, the generic questionnaires including the Pediatric Quality of Life Inventory (PedsQL) questionnaire (PedsQL™ 4.0 Generic Core Scales) and the 36-Item Short Form Health (SF-36) questionnaire are frequently used (17–20); however, the exact measures of the domains in these questionnaires have not yet been reported in the literature for patients treated with sirolimus.

The aim of this report was therefore to analyze the efficacy of sirolimus in terms of improving patients' HRQoL. The magnitude of the change (effect size) following intervention is more informative to clinical practitioners than statistically significant (whether the changes are likely to be caused by chance) but clinically unimportant changes. We, therefore, investigated the magnitude and meaningfulness of change in the PedsQL and SF-36 scores of patients with vascular malformations following a low-dose sirolimus treatment. We explored this after 6 months of treatment, which was implemented as part of the national phase IIB open-label single-arm clinical study (24). This report presents the magnitude of changes in the HRQoL of pediatric and adult patients who received doses of sirolimus with low target levels for vascular malformations. To interpret the size of the change, the results were compared with the age-adjusted norms of the Dutch population (25–28).

This report provides a detailed insight into the magnitude of changes for each domain, which are clinically relevant for the patients (and the parents of pediatric patients).

## Materials and methods

### Study design and patients

The HRQoL was measured in patients who participated in the phase IIB open-label clinical study “Treatment of Congenital Vascular Malformations Using Sirolimus: Improving Quality of Life,” which is a nationwide study performed in the Netherlands. The study is registered in ClinicalTrials.gov (identifier: NCT03987152) and EudraCT (number: 2016-002157-38). Patients with low-flow malformations included in the clinical trial had no other remaining treatment options, and the medication they had taken with the intention to relieve pain had not produced the desired effect. In total, 74 patients were enrolled at Radboud University Medical Center (Radboudumc), Nijmegen, the Netherlands, between September 2017 and February 2021. Seven patients did not complete the Challenge phase (including one case series patient), leaving a total of 67 remaining patients in the Challenge phase of the trial using low target levels of sirolimus. The patients were treated over a 6-month period (Challenge phase), which was followed by a 12-month follow-up period (Dechallenge phase). To evaluate the efficacy of the treatment, the pain was scored daily using the visual analog scale and numeric pain rating scale. In addition, magnetic resonance imaging (MRI) and HRQoL assessments were performed at the baseline and after 6 months (at end of the Challenge phase). The study demonstrated that the target levels of 4–10 ng/ml sirolimus are comparably effective and were accompanied by less severe adverse events than those reported in the literature using high target levels of 10 ng/ml or above (24).

### Outcome measures

Patients aged 2 years and older were included in the study. Two HRQoL questionnaires were used; the PedsQL questionnaire was sent as per age category (2–4 years, 5–7 years, 8–12 years, or 13–16 years) based on the age of the participant at that moment, while the SF-36 questionnaire was used for adults (17 years and older). The HRQoL questionnaires were sent out digitally before and after the 6-month treatment (Challenge phase). In all these questionnaires, higher scores indicate a better HRQoL.

### PedsQL

The PedsQL questionnaire (PedsQL™ 4.0 Generic Core Scales) is a widely used standardized generic instrument used to assess patients' and proxies' perceptions of HRQoL in pediatric patients with chronic health conditions (29, 30). This instrument contains 23 items measuring four domains of HRQoL: “Physical functioning,” “Emotional functioning,” “Social functioning,” and “School functioning.” Additionally, a “Psychosocial” score can be calculated. For each domain, a score can be calculated ranging from 0 to 100, with a higher score indicating a better HRQoL. The “total scale score” can be derived as the sum of all items over the number of items answered on all the scales (30).

### SF-36

The SF-36 questionnaire is frequently used to explore HRQoL in adults in terms of eight domains: “Physical functioning,” “Social functioning,” “Role limitations—physical problems,” “Role limitations—emotional problems,” “Mental health,” “Energy levels/vitality,” “Pain,” and “General health perception.” A mental component summary (MCS) and physical component summary (PCS) can be derived from the scores. We used the RAND-36 version 1.0, and scoring was carried out using the RAND-36 scoring guidelines (27, 28, 31). For each domain and component, a score can be derived ranging from 0 to 100, with a higher score indicating a better HRQoL.

### Statistical analyses

Statistical analyses were performed using SPSS version 25.0 (IBM). Descriptive statistics were used for the patients' demographic characteristics. When the majority of domains were normally distributed, the continuous variables and the proportions for nominal variables were presented as means with 95% confidence intervals (95% CI) or standard deviations (SD). When the majority of the domains had a skewed distribution, medians with IQR values were used.

### Comparison with the general Dutch population

The HRQoL scores of the patients were compared with the general Dutch population. For this comparison, parent PedsQL reports were used for children under 7 years of age, while the children's reports were used in children aged older than 7 years corresponding to the general Dutch population scores. HRQoL mean scores of patients with vascular malformations were compared with HRQoL mean scores of the general Dutch population. If data showed a skewed distribution, the medians of the general Dutch population were used. For children above 7 years old, these median scores are not available for the general Dutch population; therefore, mean scores of the general Dutch population were used as the median values for this group, since median scores of the general Dutch population (older than 7 years) are not published (26). Note that in large groups (as a general Dutch population) in a normal distribution, the mean value corresponds to the median value. For the children aged 2–7 years, the median scores of the general Dutch population (Parent reports) were used, as these scores of the general Dutch parent's reports under 7 are published (25).

To compare the SF36 scores of patients with vascular malformation, the published mean SF-36 scores of the general Dutch population were used (26, 27).

The difference in HRQoL between patients with vascular malformations and the general population was analyzed using a *t*-test when normally distributed or using a one-sample Wilcoxon signed-rank test when the data were skewed. All *P*-values were two-sided, and the results were considered statistically significant if *P* < 0.05.

### Changes after sirolimus treatment

The changes in HRQoL after the sirolimus treatment were analyzed. The guidelines of the particular survey were followed to calculate the correct score when values were missing. Surveys were excluded from the analysis when the surveys at the baseline and/or at the end of the Challenge phase were missing.

A change in the HRQoL of each child was quantified as a change in the total scale score of >4.4 in the self-reported PedsQL or >4.5 in the parent-reported PedsQL. These PedsQL thresholds were based on previous clinical trials within this patient category, and our phase IIB study as published previously (18, 20, 24).

In addition, the change of HRQoL per patient was quantified as a change of 3.5 in the MCS score or 4.1 in the PCS score of the SF-36 (24). The upper threshold of the minimal important difference (MID) of modest changes was used for the SF-36, based on the systematic review by Frendl et al. (32) who determined the size and meaningful changes of the SF-36 MCS and PCS and mean net of placebo changes with treatment across different diseases. This resulted in net mean modest changes of MCS [interquartile range (IQR) 0.8; 3.5] and PCS [IQR 1.6; 4.1]. The percentage of patients who experienced a change in HRQoL after 6 months of sirolimus treatment was calculated (*n* patients with a change of HRQoL/*n* of evaluable patients).

The mean differences with 95% CI between the pre-treatment (baseline) and post-treatment (Challenge phase) HRQoL scores were calculated when the differences in scores between the baseline and the end of the Challenge phase were normally distributed in the majority of domains. When these data were skewed, the median with IQR difference was calculated. For the comparison of the HRQoL scores at the baseline and the end of the Challenge phase, paired *t*-tests were used. Non-parametric tests (Wilcoxon signed rank tests) were used when the data were skewed. The threshold for statistical significance was set a priori at α = 0.05.

### Magnitude of change

The responsiveness of the HRQoL was analyzed using the widely accepted method of “effect sizes” (33–35). The effect sizes were used to translate the baseline and changes after the end of the Challenge phase into a standard unit of measurement that should provide a clearer understanding of the HRQoL results. The effect sizes can supplement standard statistical testing to obtain a more complete and clinically relevant picture of health status change (36). The effect size was calculated using Cohen's *d* formula: *d* = mean difference/SD difference. When the data were skewed, the effect size was calculated using the *z*-value (37). The effect size was considered to be small (0.20–0.49), moderate (0.50–0.79), or high (>0.80). An effect size of more than 0.5 was considered clinically relevant.

## Results

### Patient characteristics

**Figure 1:**
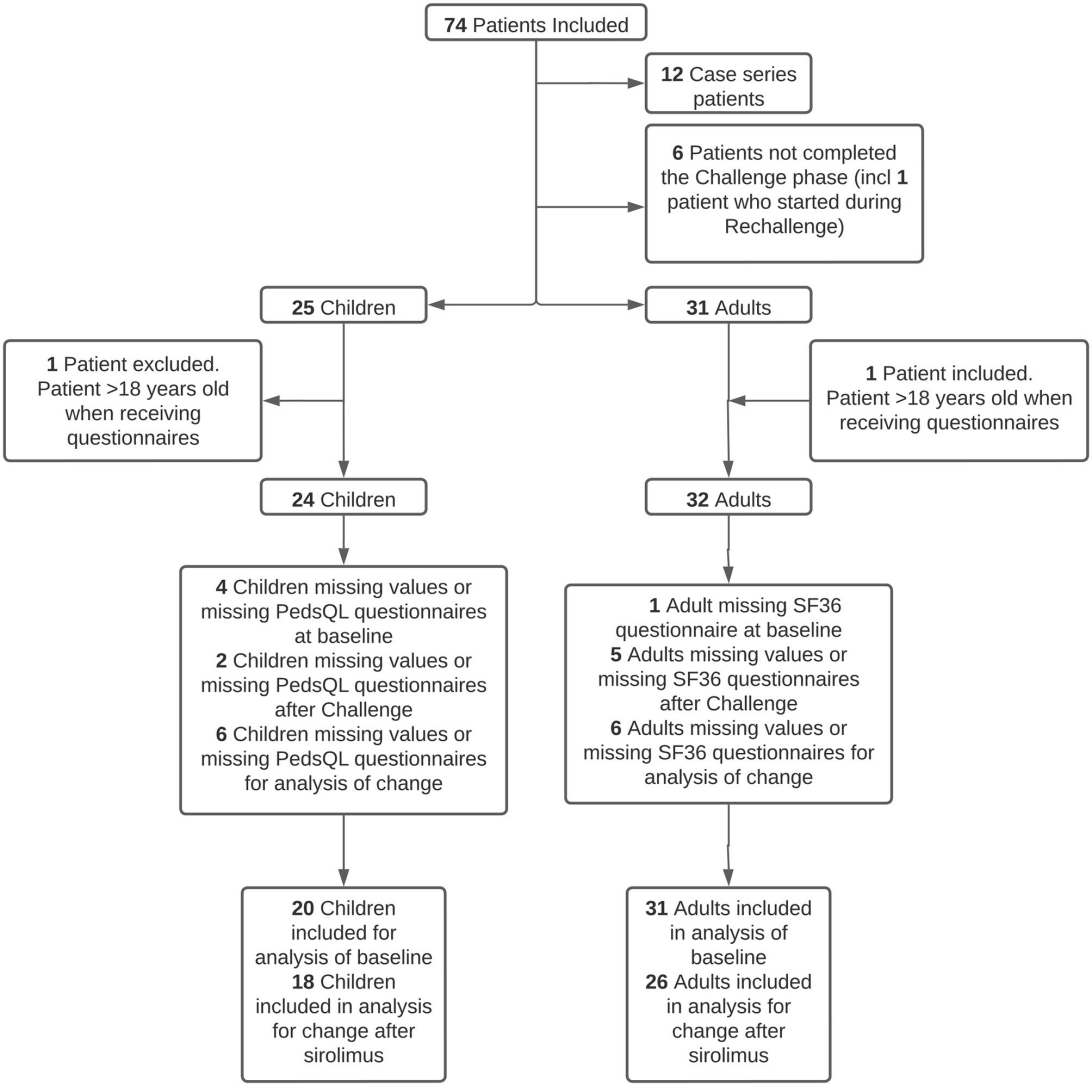
Patient distribution. In total, 12 case series patients did not receive the quality-of-life questionnaire due to different protocols (including one patient who did not complete the Challenge phase), five patients did not complete the Challenge phase, and one child was included in the study during the Rechallenge phase.

In total, 56 patients (24 children and/or parents and 32 adults) received the HRQoL surveys. At the baseline, 91.1% (*n* = 51/56) of patients (children and/or parents: 83.3%, *n* = 20/24; adults: 96.9%, *n* = 31/32) completed the surveys. The mean age of these patients was 25.8 years (SD = 16.5). Of the 56 patients, 87.5% (*n* = 49/56) of patients (children and/or parents: 91.7%, *n* = 22/24; adults: 84.4%, *n* = 27/32) completed the end of Challenge phase questionnaires. In total, 78.6% (*n* = 44/56) of patients (children: *n* = 18; adults: *n* = 26) were evaluable for the analyses of the HRQoL change after sirolimus treatment. See Figure 1 for patient distribution.

In total, 68 HRQoL questionnaires were completed (PedsQL: children [aged 5–16 years] *n* = 17 and parents [of children 2–16 years] *n* = 20; SF-36: adults *n* = 31) before the start of the sirolimus treatment (baseline). After 6 months of the sirolimus treatment, a total of 64 HRQoL questionnaires were completed (PedsQL: children [aged 5–16 years] *n* = 17 and parents [of children 2–16 years] *n* = 20; SF-36: adults *n* = 27). Despite repeated requests, data were missing for four children and one adult at the baseline, and for two children and five adults at the end of the Challenge phase. Due to this missing data before and after treatment, a total of six children and six adults were excluded from the analysis of change after the Challenge phase.

The characteristics of the included patients for the HRQoL analysis are presented in Table 1. The majority of both the pediatric and adult patients were women. At the baseline, the median total HRQoL score of the children's reports (*n* = 17) was 73.91 (IQR = 55.43; 77.17) and of the parent reports (*n* = 20) was 59.24 [IQR = 55.71; 75.54]. The means scores of Mental Component Summary (MCS) and Physical Component Summary (PCS) in adults (*n* = 31) were 47.5 [95% CI 43.4; 51.5] and 33.1 [95% CI 29.1; 37.1], respectively, at the baseline.

**Table 1:** Patient characteristics of the included patients.

**Characteristic**	**Included children (*n* = 20)**	**Included adults (*n* = 31)**
**Age groups** ***n***		
Under 2	0 (0.0%)	–
2–4	2 (10.0%)	–
5–7	3 (15.0%)	–
8–12	11 (55.0%)	–
13–17	4 (20.0%)	–
18 and older	–	31 (100%)
**Gender** ***n***		
Male	5 (25.0%)	10 (32.3%)
Female	15 (75.0%)	21 (67.7%)
**Vascular malformation type** ***n***		
Lymphatic malformation^a^	9 (45.0%)	7 (22.6%)
Venous malformation^b^	9 (45.0%)	16 (51.6%)
Combined malformation^c^	1 (5.0%)	7 (22.6%)
Other^d^	1 (5.0%)	1 (3.2%)
**Vascular malformation location** ***n***		
Head and neck	7 (35.0%)	4 (12.9%)
Thorax	0 (0.0%)	4 (12.9%)
Abdominal	1 (5.0%)	3 (9.7%)
Upper extremity	1 (5.0%)	3 (9.7%)
Lower extremity	8 (40.0%)	14 (45.2%)
Multiple locations	3 (15.0%)	3 (9.7%)

^a^Lymphatic malformation: CLOVES syndrome (*n* = 1), lymphangiomatosis (*n* = 3). ^b^Venous malformation: Blue Rubber Bleb Nevus syndrome (*n* = 1) and angio-osteohypertrophy syndrome with venous malformation (*n* = 1). ^c^Combined malformation: Klippel–Trenaunay syndrome (*n* = 7), veno-lymphatic malformation (*n* = 3), and capillary and venous malformation (*n* = 1). ^d^Other vascular malformations: fibro adipose vascular malformation (*n* = 1) and multiple spindle-cell hemangioma (*n* = 1).

### Health-related quality of life compared with the general Dutch population

**Figure 2:**
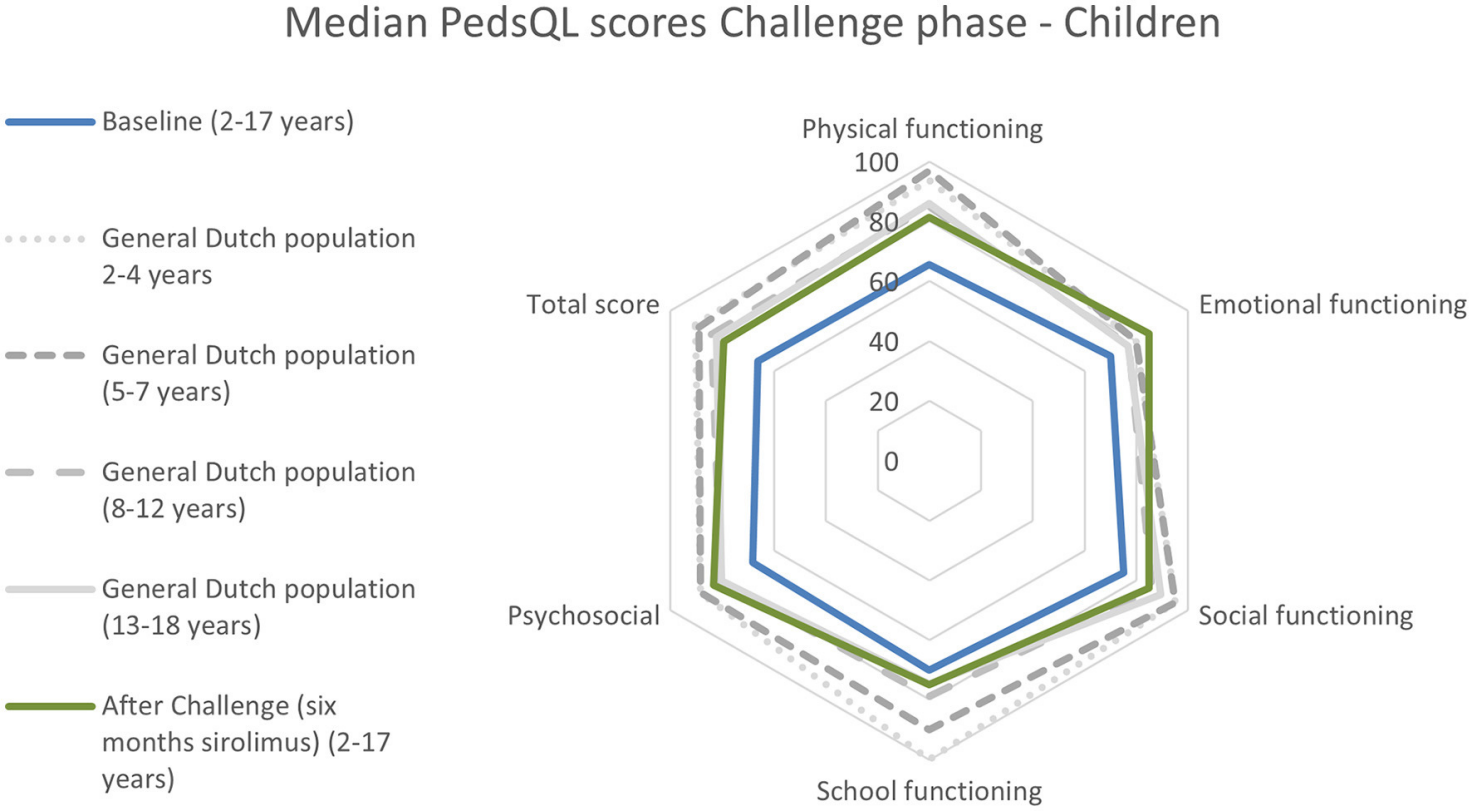
PedsQL scores of children before and after the Challenge phase for each domain. Results of the Dutch population aged 5–18 years are represented in gray (26). Baseline *n* = 19 patients with low-flow vascular malformations, and after the Challenge phase, *n* = 21 patients (parent reports for children aged 2–7 years and children's reports for patients aged 8–16 years). Exact data are presented in Supplementary Table 1. For the general Dutch population, parent reports obtained by Schepers et al. (25) are presented for children aged 2–7, while for children aged 8–16 years, the child reports obtained by van Engelen et al. (26) were used.

At the baseline, the HRQoL scores of children with vascular malformations were lower than that of the general Dutch population (Figures 1, 2, Supplementary Table 1). After the Challenge phase, the PedsQL scores of the patient group increased, and the difference in HRQoL scores between this group and the general Dutch population was smaller.

**Figure 3:**
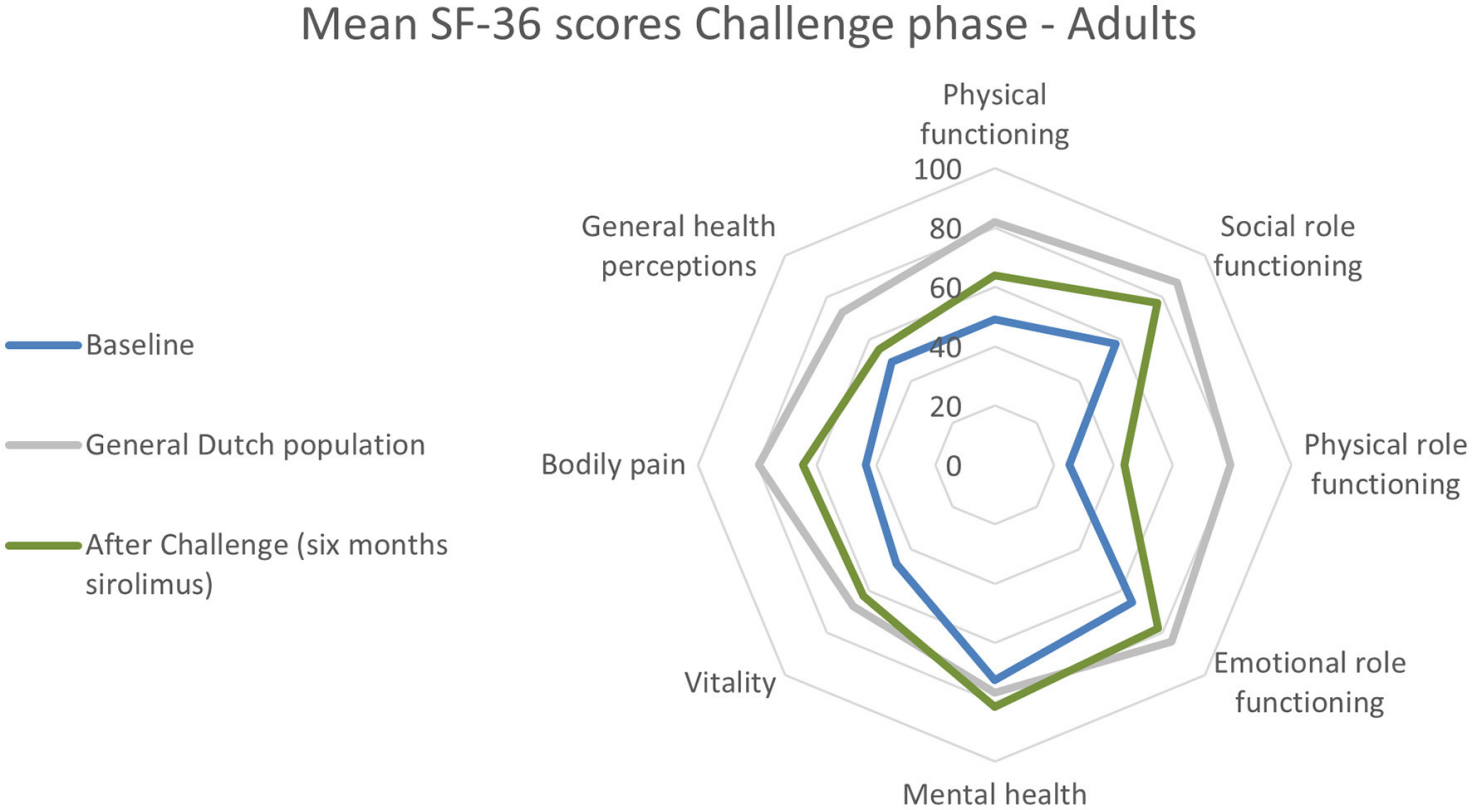
Quality-of-life scores for each domain during the Challenge phase in adults using the SF-36 questionnaire. Baseline *n* = 31 patients with low-flow vascular malformations and after Challenge phase *n* = 27. Exact data are presented in Supplementary Table 2. The RAND-36 scores for the general Dutch adult population were obtained from van der Zee et al. (27, 28).

Compared with the general Dutch population, the adult patients with vascular malformations experienced significantly lower SF-36 scores in all domains, except for “Mental health” (Figure 3, Supplementary Table 2). After the Challenge phase, the adult patient group reached the level HRQoL of the general Dutch population. The domains “Social functioning,” “Role limitations—emotional problems,” and “Energy levels/vitality” normalized after the 6-month treatment with low target levels of sirolimus.

Supplementary Tables 1, 2 show the scores of each HRQoL questionnaire in our study at the baseline and at the end of the Challenge phase compared with the general Dutch population.

### An overall change in health-related quality of life

After the Challenge phase, 29 patients had an improved HRQoL, including 77.8% of the children (*n* = 14/18) and 57.7% of the adults (*n* = 15/26). In 11.1% of the children (*n* = 2/18) and 11.5% of the adults (*n* = 3/26), a worsened HRQoL was observed.

At the baseline, the mean total scale score of the PedsQL as reported by the children (*n* = 16) was 66.3 [SD 17.7], while the parent reports (*n* = 18) gave a mean score of 64.0 [SD 16.2]. The mean total scale score of the children significantly increased by 9.9 points [SD 12.6] after the Challenge phase to 76.2 [SD 18.1], *P* < 0.05. The parental PedsQL scores showed an increase of 10.9 points [SD 10.7] after the Challenge phase to 74.9 [SD 17.0], *P* < 0.05. The PedsQL scores of each domain at the baseline and the changes after the sirolimus treatment did not significantly differ between the children and parents.

In adults (*n* = 26), the mean MCS score at the baseline was 48.9 [SD 9.3], which significantly increased to 52.5 [SD 8.7] after the Challenge phase, with a mean increase of 3.6 points ([SD 8.3], *P* < 0.005). The mean PCS score at the baseline was 32.8 [SD 10.7], which significantly increased to 39.0 [SD 13.1] after the Challenge phase, with a mean increase of 6.2 ([SD 9.5], *P* < 0.005).

Figures 2, 3 present the results of the changes in HRQoL during the Challenge phase. The detailed data are provided in Supplementary Table 3.

### Magnitude of change

**Figure 4:**
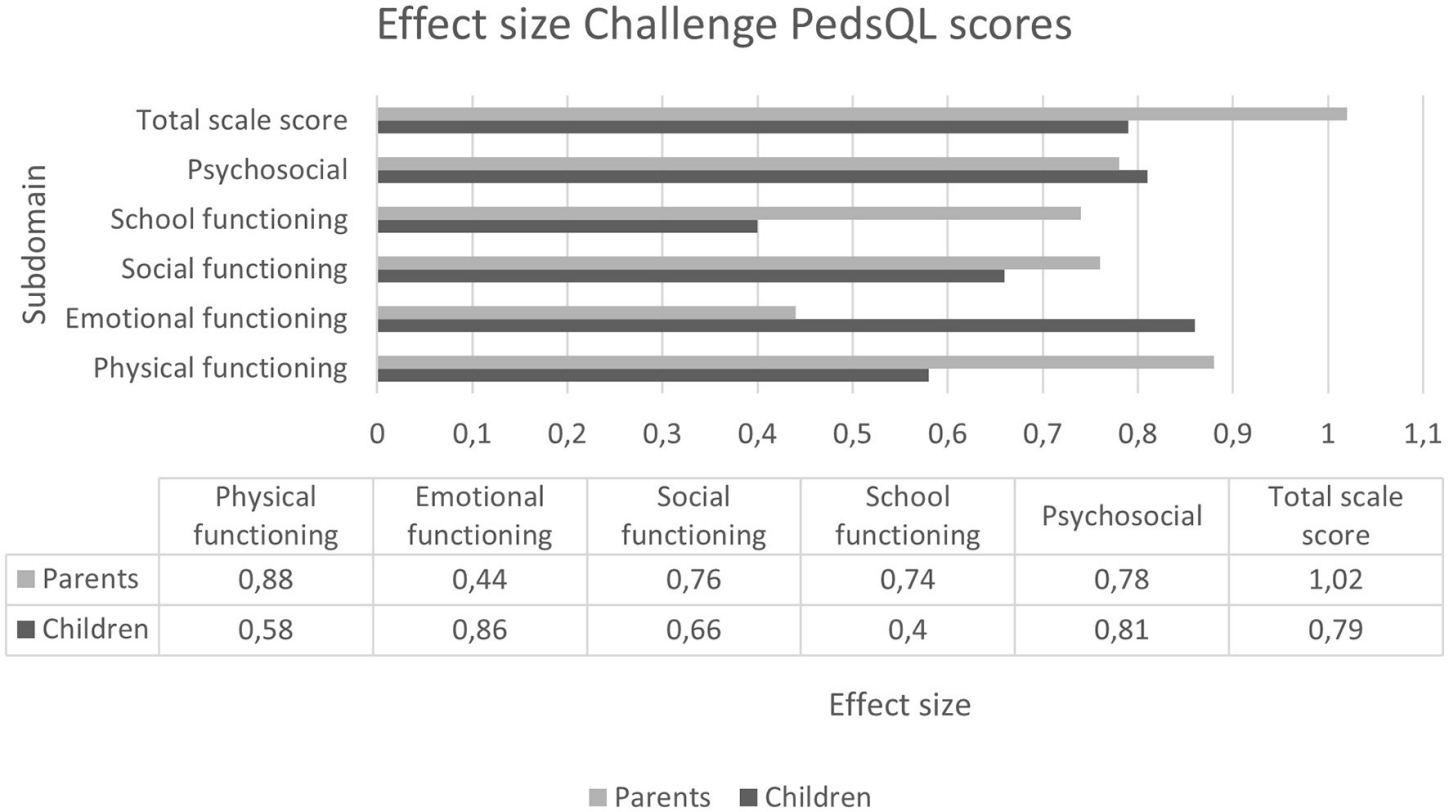
Effect sizes on each domain of the PedsQL as reported by each child (*n* = 16) and parent (*n* = 18). The effect sizes were categorized as small (0.20–0.49), moderate (0.50–0.79), or high (>0.80). An effect size of more than 0.5 is considered clinically relevant.

**Figure 5:**
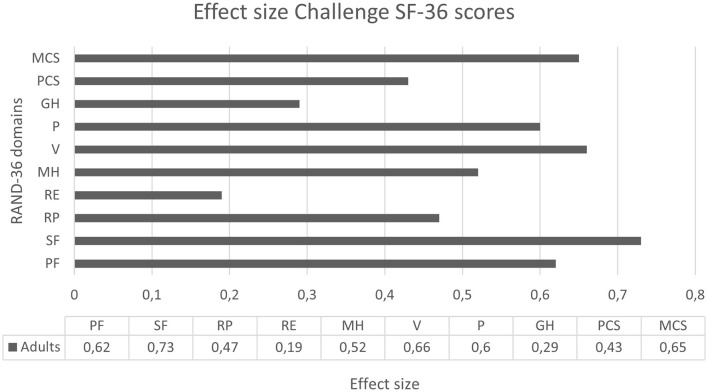
Effect sizes for the SF-36 domains at the end of the Challenge phase in 26 adult patients. The effect sizes were categorized as small (0.20–0.49), moderate (0.50–0.79), or high (>0.80). An effect size of more than 0.5 is considered clinically relevant. PF, physical functioning; SF, social functioning; RP, role limitations—physical problems; RE, role limitations—emotional problems; MH, mental health; V, energy levels/vitality; P, pain; GH, general health perception; PCS, physical component summary; and MCS, mental component summary.

The effect sizes of the sirolimus treatment on the PedsQL and SF-36 scores between the baseline and the end of the Challenge phase are shown in Figures 4, 5. In the PedsQL children's reports, a clinically relevant change was seen in all domains except for “School functioning,” in which the effect size was small (0.20–0.49). A clinically relevant moderate effect size (effect size 0.50–0.79) was seen in the domains “Physical functioning,” “Social functioning,” and “Total scores.” High (>0.80) effect sizes were observed in the domains of “Emotional functioning” and “Psychosocial functioning.” Moderate-to-high effect sizes were observed in the PedsQL parent reports in almost all domains, except “Emotional functioning.” It is noteworthy that the effect size of the “Total scale score” was 1.02. When the children's and parental reports were considered together, a clinically relevant effect size was observed in every domain.

Moderate clinically relevant effect sizes were seen in adults in the SF-36 domains “Physical functioning,” “Social functioning,” “Mental health,” “Energy levels/vitality,” “Pain,” and “Physical Component Summary.” In the other domains, a small effect size was seen.

## Discussion

The present study indicates that Dutch patients with vascular malformation experience an impaired HRQoL compared with the general population. In addition to the heterogenic group of slow-flow vascular malformations, patients have similar symptoms leading to a reduced HRQoL. All domains were significantly impaired in adults, except for “Mental health.” This result showed a worse HRQoL than that reported by Breugem et al. in patients with vascular malformations of the lower extremity, who reported that their HRQoL was not greatly impaired relative to the general Dutch population (10). Breugem et al. found impaired “Vitality” and higher levels of “Pain” in these patients; however, no differences were seen in the other dimensions of SF-36. By contrast, a systematic review of 11 studies showed that the bodily pain and mental health scores of patients with congenital vascular malformations were significantly worse than the general population of the USA, as determined using the SF-36 (38). This was also seen in a Dutch prospective cross-sectional study of 133 patients who completed the HRQoL surveys using Patient-Reported Outcome Measurement Information System (PROMIS) scales, performed by Stor et al. (39) who showed that the presence of pain negatively impacted the patients' HRQoL. In addition, the low HRQoL in our phase IIB study may be explained by the fact that the included patients had no (remaining effective) treatment options.

Because of the low number of patients per age category and the corresponding HRQoL scores of the general Dutch population, the significance of the difference in treatment effect on the HRQoL between ages was not calculated. Future research should therefore involve a larger cohort of patients to determine the changes and effect sizes in each domain for each patient age category. Additionally, it would be interesting to investigate the HRQoL effect size of different treatments, such as surgery, sclerotherapy, or (other) systemic treatments, and to compare the differences in magnitude and the meaningfulness of the changes between these treatments. In our recently published results of the clinical response to sirolimus, we showed that there was no difference in the general response to sirolimus for each vascular malformation type (24).

In the present study, we showed that the HRQoL significantly improved after the sirolimus treatment (total scale score improvement >4.4 in the self-reported PedsQL, >4.5 in the parent-reported PedsQL, >3.5 in the adult MCS score of the SF-36, and >4.1 in the PCS score of the SF-36) in 65.9% of the patients. This is comparable with the effects reported in other studies, including patients with vascular anomalies (20, 21). The SF-36 domains “Physical functioning,” “Social functioning,” “Role limitations—physical problems,” “Mental health,” “Energy levels/vitality,” and “Pain” significantly improved after 6 months of treatment with low target levels of sirolimus. Pang et al. also used the SF-36 to identify changes in each domain after 6 months of treatment with sirolimus (40). Six adult patients with low-flow head and neck vascular malformations were included and treated with sirolimus using high target levels (5–15 ng/mL). In contrast to our results, Pang et al. found no statistically significant changes in the SF-36 domains after the sirolimus treatment. The low target levels of sirolimus in our cohort (4–10 ng/mL) might play a role in this; the low occurrence of toxicities alongside the maintenance of the effectiveness of sirolimus might have resulted in a more substantially improved HRQoL (24), although more research is needed to examine this hypothesis.

A limitation of the study is that the design used in the phase IIB clinical trial did not include a placebo group. The optimal design would be a randomized placebo clinical trial (RCT); however, to prove the efficacy of sirolimus in their HRQoL, the concept of Challenge, Dechallenge, and Rechallenge (CDR design) can be used. This CDR design, in which the patients are under their own control, is frequently used for rare diseases for *N*-of-1 clinical trials to assess efficacy (24, 41).

Other studies investigated the change in HRQoL after treatment; however, they did not analyze or show the number of patients who experienced changes in their HRQoL and/or their specific changes in each domain after treatment in both children and adults (18, 20, 21, 39, 42).

Lokhorst et al. developed recently a new questionnaire, the OVAMA questionnaire, which could be additionally used in future research (43). This is a disease-specific questionnaire, which is not suitable for comparing the HRQoL scores with the general (Dutch) population and/or other (chronic) diseases. The effect of sirolimus was not investigated in the study by Lokhorst et al., which might have influenced the clinical improvements that patients showed and the ability to pick up improvements using the SF-36. Additionally, the SF-36 can be used to calculate a utility score, which is necessary for a cost-effective analysis. For these reasons, the additional use of generic questionnaires such as SF-36 remains necessary.

## Conclusion

This study shows that adult and pediatric patients with vascular malformation experience a decreased HRQoL compared with the general Dutch population. Six months of treatment with low target levels of sirolimus significantly improved the HRQoL in adults, while in children, a clear tendency to improve was also observed. This study is one of the first studies to investigate the magnitude of change in the HRQoL resulting from sirolimus treatment in patients with vascular malformations. Sirolimus led to a moderate-to-high clinically relevant change in multiple domains in the adult population. In conclusion, the health-related quality of life of children and adults with vascular malformations improved after treatment with low target levels of sirolimus, leading to a more normalized HRQoL.

## Author's note

CV and LS are part of Project ID: 769036 within the Members of the Vascular Anomalies Working Group (VASCA WG) of the European Reference Network for Rare Multisystemic Vascular Diseases (VASCERN).

## Data availability statement

The data that support the findings of this study are available from the corresponding author upon reasonable request.

## Ethics statement

The studies involving human participants were reviewed and approved by Central Committee on Research Involving Human Subjects (CCMO). Written informed consent to participate in this study was provided by the participants' legal guardian/next of kin.

## Author contributions

VH conducted the clinical trial, drafted the manuscript, and analyzed and interpreted the data. FB and IR conducted the clinical trial and critically reviewed the manuscript. BV, CV, LS, PL, CH, and WK critically reviewed the manuscript. WK analyzed the data. DL is the principal investigator of the clinical trial, critically reviewed the analysis, interpreted the data, and significantly contributed to writing the manuscript. All authors read and approved the final manuscript.
