# Supplementary material for: Magnitude and relevance of change in health-related quality of life in patients with vascular malformations treated with sirolimus
Source: Front Med (Lausanne). 2023 Apr 20;10:1155476. doi: 10.3389/fmed.2023.1155476 (PMC10157393; doi:10.3389/fmed.2023.1155476)
Supplement: Supplementary file 1 [file Table_1.DOCX]

Supplementary Material

Magnitude and relevance of change in health-related quality of life in patients with vascular malformations treated with sirolimus

Sirolimus and HRQoL in VM

Veroniek E.M. Harbers^1,2^, MD, Frédérique C.M. Bouwman^2,3^, MD, Ingrid M.P. van Rijnsoever^2,4^, Bas H. Verhoeven^2,3^, MD, Carine J.M. van der Vleuten^2,5,6^, MD, Leo J. Schultze Kool^1,2,6^, MD, Peter C.J. de Laat^7^, MD, Chantal M.A.M. van der Horst^8^, MD, Wietske Kievit^9^, Maroeska D.M.W.M. te Loo^2,6,10*^, MD

*** Correspondence:** Corresponding Author: [Maroeska.teloo@radboudumc.nl](mailto:Maroeska.teloo@radboudumc.nl)

##

## Supplemental Table 1. PedsQL scores in child patients compared with the general Dutch population

| **Population age group** | **PedsQL scores in the vascular malformation population at the baseline** | **PedsQL scores in the vascular malformation population at the end of Challenge phase** | **General Dutch population sample** |
| --- | --- | --- | --- |
| **Children aged 2–4 years** – Parents report | Baseline, n = 2,  median (IQR) | End of Challenge phase,  n = 3, median (IQR) | General Dutch population sample median, n = 275 |
| PF | 42.19 (9.38 – NA) | 81.25 (37.50 – NA) | 93.75 |
| EF | 67.50 (65.0 – NA) | 75.00 (55.0 – NA) | 80.0 |
| SF | 52.50 (45.0 – NA) | 50.00 (45.0 – NA) | 95.0 |
| Sc.F | NA | 83.33 (n = 1) | 100.0 |
| PS | 60.00 (57.5 – NA) | 62.50 (50.0 – NA) | 88.46 |
| Total scale score | 52.08 (38.89 – NA) | 70.83 (44.44 – NA) | 90.48 |
| **Children aged 5–7 years** – Parents report | Baseline n = 3,  median (IQR) | End of Challenge n = 3, median (IQR) | General Dutch population sample median, n = 251 |
| PF | 56.25 (40.63 – NA) | 81.25 (59.38 – NA) | 96.88 |
| EF | 75.00 (70.00 – NA) | 85.00 (75.00 – NA) | 80.0 |
| SF | 75.00 (65.00 – NA) | 90.00 (75.00 – NA) | 95.0 |
| Sc.F | 65.00 (55.0 – NA) | 80.00 (75.00 – NA) | 90.0 |
| PS | 71.67 (63.33 – NA) | 85.00 (83.33 – NA) | 88.33 |
| Total scale score | 66.30 (55.43 –  NA) | 83.70 (75.00 – NA) | 89.13 |
| **Children aged 8–12 years** – Child report | Baseline n = 10,  median (IQR) | End of Challenge  n = 10, median (IQR) | General Dutch population sample n = 219, mean = median |
| PF | 60.94 (43.75 – 89.06) | 81.25 (68.75 – 93.75) | 84.87 (9.3) |
| EF | 67.50 (53.75 – 77.50) | 85.00 (80.00 – 90.00) | 77.05 (13.66) |
| SF | 75.00 (58.75 – 87.50) | 80.00 (60.00 – 95.00) | 86.14 (12.3) |
| Sc.F | 70.00 (53.75 – 81.25) | 75.00 (60.00 – 80.00) | 78.7 (12.0) |
| PS | 69.2 (57.50 – 81.25) | 78.33 (68.33 – 88.33) | 80.63 (10.31) |
| Total scale score | 69.02 (53.80 – 79.89) | 79.35 (65.22 – 90.22) | 84.18 (8.87) |
| **Children aged 13–16 years** – Child report | Baseline n = 4,  median (IQR) | End of Challenge  n = 3, median (IQR) | General Dutch population (13–18 years) n = 185,  sample mean (SD) |
| PF | 70.31 (54.69 – 88.28) | 82.81 (75.78 – 92.19) | 86.01 (9.77) |
| EF | 70.00 (60.00 – 83.75) | 77.50 (51.25 – 100.00) | 76.7 (15.2) |
| SF | 90.00 (86.25 – 93.75) | 87.50 (81.25 – 93.75) | 89.38 (11.56) |
| Sc.F | 70.00 (43.75 – 85.00) | 60.00 (56.25 – 71.25) | 74.59 (13.16) |
| PS | 75.83 (65.83 – 84.58) | 75.83 (65.83 – 84.58) | 80.23 (10.18) |
| Total scale score | 78.26 (69.84 – 86.68) | 78.26 (69.84 – 86.68) | 82.24 (9.15) |
| **All children aged 2–16 years –** Parent and child report | Baseline n = 19,  median (IQR) | End of Challenge n = 21 median (IQR) | General Dutch population* |
| PF | 65.63 (46.88 – 84.38) | 81.25 (70.31 – 90.63) | Unknown |
| EF | 70.00 (60.00 – 80.00) | 85.00 (65.00 – 92.50) | Unknown |
| SF | 75.00 (60.00 – 90.00) | 85.00 (67.50 – 92.50) | Unknown |
| Sc.F | 70.00 (55.00 – 82.50) (n=17) | 75.00 (60.00 – 80.00) (n=19) | Unknown |
| PS | 68.33 (61.67 – 80.00) | 83.33 (66.67 – 87.50) | Unknown |
| Total scale score | 66.30 (55.43 – 77.17) | 79.35 (70.11 – 89.13) | Unknown |

## **No known data for children of the general Dutch population aged 2–16 years. Median quality of life scores of parent reports from the general Dutch population were compared with the PedsQL scores of the patients’ parents (children aged 2–7 years)(1). For the age groups 8–16 years, the mean PedsQL scores from the child reports were used as the median value (2). ^1^Median scores of the parent reports were used for children aged 2–7 years, and mean scores of the child reports were used for children aged 8–16 years.*

## *Abbreviations: EF: Emotional functioning; NA: not applicable; PF: Physical functioning; PS: calculated Psychosocial score; SF: Social functioning; Sc.F: School functioning.*

## Supplemental Table 2. SF-36 scores in adult patients compared with the general Dutch population

| **Population** | **SF-36 scores in the low-flow vascular malformation population** | | | | **General Dutch population sample** |
| --- | --- | --- | --- | --- | --- |
| Domain | Baseline  n = 31 | Mean difference compared to Dutch population^1^ | End of Challenge n = 27 | Mean difference compared to Dutch population^1^ | N = 1063 |
| PF mean, (95%CI) | 49.0  (39.4 – 58.7) | P<0.005* | 63.9  (50.9 – 76.8) | P<0.05* | 81.9 |
| SF mean, (95%CI) | 57.7  (47.0 – 68.4) | P<0.005* | 77.3  (67.2 – 87.4) | P>0.05 | 86.9 |
| RP mean, (95%CI) | 25.0  (12.9 – 37.1) | P<0.005* | 43.5  (25.6 – 61.4) | P<0.005* | 79.4 |
| RE mean, (95%CI) | 65.6  (50.6 – 80.6) | P<0.05* | 77.8  (61.8 – 93.7) | P>0.05 | 84.1 |
| MH mean, (95%CI) | 72.5  (67.0 – 78.0) | P>0.05 | 81.6  (76.5 – 86.8) | P>0.05 | 76.8 |
| V mean,  (95%CI) | 47.1  (40.9 –53.3) | P<0.005* | 62.6  (53.8 – 71.4) | P>0.05 | 67.4 |
| P mean,  (95%CI) | 43.4  (33.1 – 53.6) | P<0.005* | 64.8  (53.7 – 75.8) | P<0.05* | 79.5 |
| GH mean, (95%CI) | 49.0  (40.7 – 57.3) | P<0.005* | 55.0  (46.4 – 63.6) | P<0.005* | 72.7 |
| PCS mean, (95%CI) | 33.1  (29.1 – 37.1) | NA | 39.6  (34.4 – 44.8) | NA | UNK |
| MCS mean, (95%CI) | 47.5  (43.4 – 51.5) | NA | 52.8  (49.4 – 56.2) | NA | UNK |

*Table 2. RAND 36-item Health Survey 1.0 scale scores were computed by summing item scores and transforming them into a 100-point scale, “0” refers to the worst health and “100” the best health. General Dutch population sample (3, 4). Mean scores of the SF-36 (RAND-36) from the general Dutch population was used as the median score and compared with Dutch patients with low-flow vascular malformations (3). ^1^t-tests *Significant difference to the General Dutch population.*

*Abbreviations: GH: General health perception; HC: Health change; IQR: interquartile range; MH: Mental health; NA: not applicable; P: Pain; PF: Physical functioning; RE: Role limitations - emotional problems; RP: Role limitations - physical problems; SF: Social functioning; V: Energy levels/vitality; UNK: unknown*.

**Supplemental Table 3. HRQoL results during the Challenge phase, including the differences and effect sizes**

| **Questionnaires** | **Baseline score** | **End of Challenge score** | **Differences between phases** | |
| --- | --- | --- | --- | --- |
| **Children’s report, n = 16** | | | | |
| **Domain** | **Mean (SD)** | **Mean (SD)** | **Differences in Mean (SD, *p*)^1^** | **Effect size^2^** |
| Physical functioning | 65.2 (22.3) | 76.4 (22.2) | 11.1 (19.0, P<0.05*) | 0.58 |
| Emotional functioning | 65.3 (20.4) | 76.3 (21.3) | 10.9 (12.7, P<0.005*) | 0.86 |
| Social functioning | 72.8 (21.8) | 82.5 (20.4) | 9.7 (14.8, P<0.05*) | 0.66 |
| School functioning | 62.5 (18.0) | 69.7 (17.6) | 7.2 (17.8, P>0.05) | 0.40 |
| Psychosocial | 66.9 (17.6) | 76.1 (17.3) | 9.3 (11.5, P<0.05*) | 0.81 |
| Total scale score | 66.3 (17.7) | 76.2 (18.1) | 9.9 (12.6, P<0.05*) | 0.79 |
| **Parent report, n = 18** | | | | |
| **Domain** | **Mean (SD)** | **Mean (SD)** | **Differences in Mean (SD, *p*)^1^** | **Effect size^2^** |
| Physical functioning | 63.7 (22.2) | 77.1 (17.9) | 13.4 (15.3, P<0.005*) | 0.88 |
| Emotional functioning | 63.3 (21.4) | 70.6 (21.9) | 7.2 (16.3, P>0.05) | 0.44 |
| Social functioning | 67.2 (17.8) | 77.8 (20.7) | 10.6 (13.9, P<0.05*) | 0.76 |
| School functioning** | 63.1 (18.2) | 74.7 (19.9) | 11.6 (15.7, P<0.05*) | 0.74 |
| Psychosocial | 64.4 (16.6) | 73.7 (18.2) | 9.2 (11.8, P<0.005*) | 0.78 |
| Total scale score | 64.0 (16.2) | 74.9 (17.0) | 10.9 (10.7, P<0.005*) | 1.02 |
| **Age group 18 years and older, n = 26** | | | | |
| **Domain** | **Mean (SD)** | **Mean (SD)** | **Differences in Mean (SD, *p*)^1^** | **Effect size^2^** |
| Physical functioning | 49.4 (26.6) | 62.5 (32.6) | 13.1 (21.0, P<0.005*) | 0.62 |
| Social functioning | 58.2 (28.9) | 76.4 (25.6) | 18.3 (25.1, P<0.005*) | 0.73 |
| Role limitations - physical problems | 23.1 (31.6) | 41.3 (44.7) | 18.3 (39.1, P<0.05*) | 0.47 |
| Role limitations - emotional problems | 69.2 (38.8) | 76.9 (40.8) | 7.7 (41.4, P>0.05) | 0.19 |
| Mental health | 75.2 (12.4) | 80.9 (12.8) | 5.7 (10.9, P<0.05*) | 0.52 |
| Energy levels/vitality | 48.5 (16.3) | 61.3 (21.8) | 12.9 (19.4, P<0.005*) | 0.66 |
| Pain | 44.7 (27.3) | 63.4 (27.6) | 18.7 (31.4, P<0.005*) | 0.60 |
| General health perception | 50.0 (23.4) | 54.4 (22.0) | 4.4 (15.1, P>0.05) | 0.29 |
| Mental component summary | 48.9 (9.3) | 52.5 (8.7) | 3.6 (8.3, P<0.05*) | 0.43 |
| Physical component summary | 32.8 (10.7) | 39.0 (13.1) | 6.2 (9.5, P<0.005*) | 0.65 |

*The effect sizes were categorized as small (0.20 – 0.49), moderate (0.50 – 0.79), and high (>0.80).^1^Paired samples t-tests. ^2^Effect size calculation using Cohen’s d formula: d = mean difference/SD difference. *Significant difference. **n = 16, two patients of 2–4 years at the baseline.*

1. Schepers SA, van Oers HA, Maurice-Stam H, Huisman J, Verhaak CM, Grootenhuis MA, et al. Health related quality of life in Dutch infants, toddlers, and young children. Health Qual Life Outcomes. 2017;15(1):81.

2. Engelen V, Haentjens MM, Detmar SB, Koopman HM, Grootenhuis MA. Health related quality of life of Dutch children: psychometric properties of the PedsQL in the Netherlands. BMC Pediatr. 2009;9:68.

3. VanderZee KI, Sanderman R, Heyink JW, de Haes H. Psychometric qualities of the RAND 36-Item Health Survey 1.0: a multidimensional measure of general health status. Int J Behav Med. 1996;3(2):104-22.

4. Zee KI van der SR. Het meten van de algemene gezondheidstoestand met de RAND-36, een handleiding. Tweede herziene druk. UMCG / Rijksuniversiteit Groningen, Research Institute SHARE. 2012.
